# A Real-Time Fault Diagnosis Method for Multi-Source Heterogeneous Information Fusion Based on Two-Level Transfer Learning

**DOI:** 10.3390/e26121007

**Published:** 2024-11-22

**Authors:** Danmin Chen, Zhiqiang Zhang, Funa Zhou, Chaoge Wang

**Affiliations:** 1 School of Computer and Artificial Intelligence, Henan Finance University, Zhengzhou 450046, China; chendmhenu@163.com; 2 Zhengzhou Key Laboratory of Financial Big Data Intelligent Application Technology, Zhengzhou 450046, China; 3 School of Logistic Engineering, Shanghai Maritime University, Shanghai 201306, China; zqiang_zhang@foxmail.com (Z.Z.); cgwang@shmtu.edu.cn (C.W.)

**Keywords:** real-time fault diagnosis, transfer learning, information fusion

## Abstract

A convolutional neural network can extract features from high-dimensional data, but the convolution operation has a high time complexity and requires a large amount of computation. For equipment with a high sampling frequency, fault diagnosis methods based on convolutional neural networks cannot meet the requirements of online fault diagnosis. To solve this problem, this study proposes a fault diagnosis method for multi-source heterogeneous information fusion based on two-level transfer learning. This method aims to fully utilize multi-source heterogeneous information and external domain data, construct a two-level transfer mechanism to fuse multi-source heterogeneous information, avoid convolutional operations, and achieve real-time fault diagnosis. Its main work is to build a feature extraction network model of screenshots, design a mechanism for transfer from the feature extraction model using screenshots to the deep learning model using one-dimensional sequence signals, and complete the transfer from a convolutional neural network to a deep neural network. After two-level transfer, the fault diagnosis model not only integrates the characteristics of one-dimensional sequence signals and screenshots but also avoids convolution operations and has a low time complexity. The effectiveness of the proposed method is verified using a gearbox dataset and a bearing dataset.

## 1. Introduction

Deep learning is an effective data feature extraction technology, and conducting research on fault diagnosis methods based on deep learning can help promote the implementation of artificial intelligence technology in the field of intelligent manufacturing equipment diagnosis [1,2]. The fault diagnosis models based on deep learning mainly include Deep Belief Networks (DBNs), convolutional neural networks (CNNs), Recurrent Neural Networks (RNNs), and stacked autoencoders (SAEs). The DBN can train models layer by layer through unsupervised pre-training to capture high-level abstract features in data, but it ignores the importance of local information for fault diagnosis [3,4]. The RNN, due to its unique memory capabilities and parameter-sharing characteristics, can capture autocorrelation information of time series data, but its global feature extraction ability is insufficient [5]. The fault diagnosis method based on the SAE has attracted widespread attention from experts in the field of fault diagnosis due to its simple structure and strong feature extraction ability. However, the SAE’s processing of high-dimensional data can cause parameter dilation, while CNNs have achieved remarkable success in the field of fault diagnosis due to their unique structural advantages, as it has no pressure to process high-dimensional data through shared convolution kernels.

The effectiveness of deep learning depends on the quantity, quality, and utilization of data. The data structure collected in industrial sites may involve single types of data or different types of data collected through different types of sensors, such as accelerometers and cameras. Common storage forms include one-dimensional sequences, two-dimensional images, or video surveillance. Different types of data contain different types of features, which will bring certain difficulties in fault diagnosis when using heterogeneous data because different neural network models have certain advantages for certain data structures, but a single network model cannot effectively utilize the correlations and complementarity in different storage forms of data. Therefore, utilizing the correlation and fusion of heterogeneous data to maximize the value of data from the perspective of data utilization has become an important issue in industrial big data analysis [6].

The conclusion of [7] shows that research on intelligent fusion can combine all possible collected data or all possible features extracted using different methods and promote the development of mechanical fault diagnosis. The authors of [8,9,10,11,12,13,14,15,16,17] fully integrate multi-source heterogeneous information to improve the accuracy of fault diagnosis. A hybrid learning algorithm for gearbox fault diagnosis based on a multi-layer perceptron (MLP) and a convolution neural network (CNN) is proposed in [8]. A bearing fault diagnosis model integrating deep neural networks (DNNs) and a CNN is proposed in [9]. CNN and DNN models extract different features from their respective datasets and fuse these features for fault diagnosis. In order to improve the robustness to background noise and the diagnosis performance when analyzing damaged sensor readings, the authors of [10] proposed a multi-sensor data fusion framework for mechanical fault diagnosis based on a thermal imager and vibration monitoring, and they developed a decision-level end-to-end fault diagnosis method based on deep learning and weighted Dempster–Shafer evidence theory. In order to further improve the effect of multi-sensor information fusion, the authors of [11] proposed a new multi-sensor information integration fault diagnosis method based on ensemble learning. The authors of [12] proposed a diagnostic method based on multiple information fusion technology and an unsupervised representation alignment deep Q network. The authors of [13] proposed a deep residual CNN with an enhanced feature learning ability and information fusion ability for multi-task bearing fault diagnosis. This method transformed and integrated multi-sensor data with domain knowledge to construct an information map. The ability to learn recognition features was improved by introducing two attention modules. The authors of [14] proposed a gear fault diagnosis method based on information fusion and a CNN. The power spectral density (PSD) of the original signal collected by multiple sensors was calculated to obtain the frequency-domain signal, which was converted into a PSD energy map after information fusion and PSD analysis. The obtained energy map was input into VGG16 as a sample for training and diagnosis. The authors of [15] proposed a method for diagnosing composite faults in rolling bearings using multi-sensor decision fusion and convolutional neural networks. The authors of [16] proposed a multi-sensor residual convolution fusion network that extracts parallel extraction of local key features and global fusion of vibration and acoustic sensor data. In order to consider the distribution gap and intrinsic correlations between multi-source mechanical signals, the authors of [17] proposed a cross-modal fusion convolutional neural network for mechanical fault diagnosis that performs mode-specific and cross-modal feature representation on multi-source data.

The above studies were able to achieve high fault diagnosis accuracy, but there are two problems. Firstly, fault diagnosis methods based on CNNs require a large number of fault information images, and training CNNs is very time-consuming. Secondly, fault diagnosis methods based on CNNs require many convolution operations to obtain fault image features and then output the diagnosis results according to the features. However, the time complexity of convolution operations is high. For equipment with a high sampling frequency, a fault diagnosis method based on a CNN cannot meet the requirements of online fault diagnosis. 

Both fault images and other images are composed of diagonal lines, color boundaries, circles, and stripes. The method of using an existing CNN trained on large datasets to improve the accuracy of fault diagnosis models and reduce the training time is particularly important [18]. Transfer learning is a machine learning method that migrates the data or features of one domain (source domain) to another domain (target domain) and uses the source domain with more data or features to improve the model performance of the target domain with fewer data or features [19,20,21]. Transfer learning has made great progress in the image field [22], natural language processing [23], medical health [24], and fault diagnosis [25,26,27,28,29,30,31,32,33,34,35,36]. Fault diagnosis methods based on transfer learning can be divided into two categories: domain adaptation [25,26,27,28,29,30,31,32,33] and model transfer [34,35,36]. 

Due to the different probability distributions of data in the source and target domains, domain adaptation is used to make the distributions of the source and target domains as close as possible. The authors of [25] proposed a deep multi-source transfer learning model using a new source domain, Wasserstein distance output alignment classifier, and weighted classifier. The authors of [26] proposed a novel deep imbalanced domain adaptation framework for the fault diagnosis of bearings, aiming at a challenging scenario where feature shift and label shift exist simultaneously under different working conditions. Considering the practical issue of extremely limited fault data in machinery fault diagnosis, the authors of [27] proposed a novel network by simultaneously conducting supervised classification and multiple adversarial domain adaptation to improve the performance of deep transfer learning. In order to consider the distribution of each category when matching the global distribution of source domain features and target domain features in the process of obtaining domain invariant features, a class-level matching transfer learning network was proposed in [28]. The authors of [29] proposed a deep transfer learning method that minimizes inter-domain decision differences. The proposed method directly measures and minimizes the differences in the decision result matrix to promote the minimization of distribution differences between two domain data. The authors of [30] proposed a novel cross-domain fusion network for multi-modal data that utilizes vibration signals and thermal images to capture comprehensive information about the health status of the gearbox. Firstly, one-dimensional and two-dimensional convolutional neural networks are constructed for feature extraction and fusion of multi-modal data. Then, the maximum mean difference loss is introduced to achieve cross-domain feature alignment within the modality. Finally, a cross-modal consistency learning strategy was constructed to improve the cross-domain diagnostic performance of the model. The authors of [31] proposed a new framework for the cross-domain fault diagnosis of rotating machinery. The proposed method provided a unified network structure for aligning feature representations to improve knowledge transfer from labeled vibration signals (source domain) to unlabeled vibration information (target domain). The authors of [32] introduced a new approach called cross-domain augmentation (CDA) for diagnosing unseen working conditions that often occur in industrial scenarios. To achieve this idea, an adversarial-domain-enhanced generalization (ADAG) method was proposed, which enhanced the domain through a convex combination of data and feature labels. The authors of [33] proposed a digital-twin-driven fault diagnosis framework for achieving fault diagnosis of rolling bearings in the absence of sufficient training data. Firstly, a dynamic virtual representation method for rolling bearings was established to generate simulated data. Then, a transformer-based network was developed to learn knowledge of simulated data for diagnosis. Meanwhile, a selective adversarial strategy was introduced to achieve cross-domain feature alignment when the health status of the measurement data is unknown.

Model transfer is a common strategy for training deep models in cross-domain scenarios in various applications. The intuitive idea of model transfer is that a pre-processed deep neural network is trained on a source domain with a large amount of labeled data and then fine-tuned with a small amount of target data on a pre-trained deep neural network. The authors of [34] proposed a bearing fault diagnosis model based on the transfer AlexNet network, which only requires replacing the last fully connected layer, thereby reducing the sample size and training time when establishing a new model. The authors of [35] proposed a fault diagnosis model based on deep transfer learning. Using the VGG16 network trained on the ImageNet dataset, the general features of the first three convolutional layers were transferred through model transfer, and then the last two convolutional layers and fully connected layers were fine-tuned using faulty time–frequency images. The authors of [36] proposed a transfer learning convolutional neural network based on AlexNet for bearing fault diagnosis. Firstly, the original vibration signal is reconstructed into a signal within the fault resonance frequency band using a frequency-sliced wavelet transform, resulting in a two-dimensional time–frequency image of the reconstructed signal. Secondly, the AlexNet network is migrated, specifically by transferring the parameters of the first five layers from the pre-trained AlexNet network and fine-tuning them to adapt to the bearing fault dataset. Finally, the features of the two-dimensional time–frequency images are extracted using the fine-tuned model, achieving the classification of bearings with faster training speed and higher accuracy.

**Figure 1:**
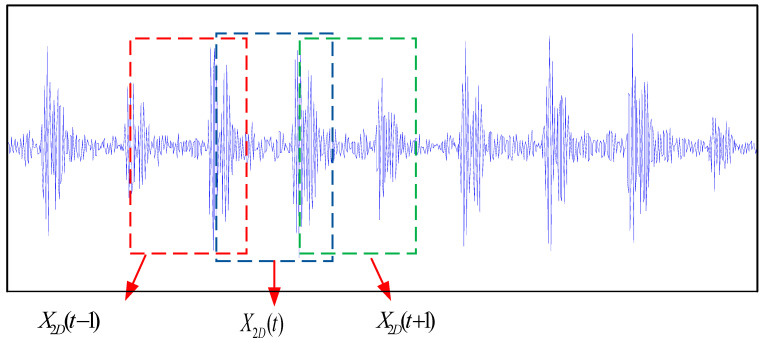
Two-dimensional screenshot diagram.

In actual device monitoring, the center monitor is used to present dynamic change curves of values collected by certain key sensors. These monitoring screenshots can show the amplitude and trend of vibration signals over a period of time, as shown in Figure 1. Two-dimensional images can intuitively reflect the trend information of signals, making up for the shortcomings of extracting the overall features of one-dimensional signals. If only one type of heterogeneous data from multiple sources is used for deep learning fault diagnosis, it will inevitably result in the waste of information contained in other data, which will affect the accuracy of fault diagnosis. Convolutional neural networks are commonly used to extract fault features from high-dimensional data in multi-source heterogeneous data. However, fault diagnosis methods based on CNNs require a large number of fault information images, and training CNNs is very time-consuming. Whether they are fault images or other images, they are composed of abstract concepts such as diagonals, color boundaries, circles, and stripes. It is particularly important to utilize existing CNNs trained on large datasets to improve the accuracy of fault diagnosis models and reduce training time. However, existing transfer-learning-based convolutional neural network models have multiple convolutional layers and high computational complexity. For devices with high sampling frequencies, fault diagnosis methods based on convolutional neural networks cannot meet the requirements of online fault diagnosis.

In order to improve the accuracy of fault diagnosis by fusing the features of two-dimensional images and avoid convolution operations to improve the real-time performance of fault diagnosis, this study proposes a real-time fault diagnosis method for multi-source heterogeneous information fusion based on two-level transfer learning (TTDNN). This method integrates multi-source heterogeneous information through two-stage migration and avoids convolution operations, achieving real-time fault diagnosis.

The main contributions of this study can be summarized as follows:

1. The mechanism of transfer from a feature extraction model of screenshot images to a deep learning model of one-dimensional sequence signals is designed, and the transfer from a CNN to a DNN is realized.
2. Aiming at the problem of poor real-time performance of fault diagnosis algorithms when multi-source heterogeneous information and external domain data are fully utilized, a real-time fault diagnosis method for multi-source heterogeneous information fusion based on two-level transfer learning is proposed. The purpose of this method is to make full use of multi-source heterogeneous information and external domain data, build a two-level transfer mechanism to fuse multi-source heterogeneous information, avoid convolution operations, and achieve the purpose of real-time fault diagnosis.

The rest of this article is organized as follows. Section 2 introduces the fault diagnosis method for multi-source heterogeneous information fusion based on two-level transfer learning in detail. Section 3 presents a time complexity analysis. Section 4 describes the experiment and analysis using gearbox and bearing datasets. Finally, Section 5 summarizes this study and provides future research directions.

## 2. Proposed Method 

**Figure 2:**
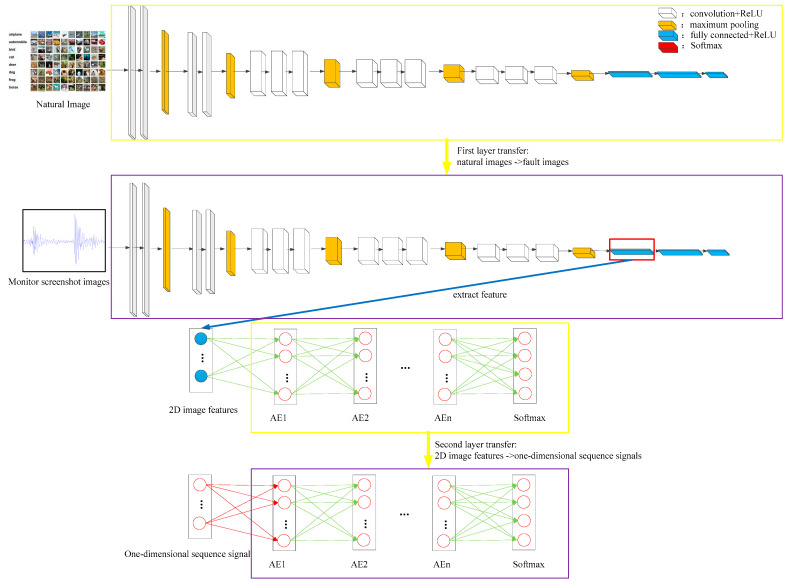
Fault diagnosis framework of multi-source heterogeneous information fusion based on two-level transfer learning.

Fault diagnosis with multi-source heterogeneous information fusion based on two-level transfer learning is divided into two stages: fault diagnosis model construction with multi-source heterogeneous information fusion based on two-level transfer learning and online fault diagnosis. The construction stage of the multi-source heterogeneous information fusion fault diagnosis model based on two-level transfer learning is shown in Figure 2. Both fault images and natural images are composed of abstract concepts such as diagonals, color boundaries, circles, and stripes. Existing CNNs trained on large natural image datasets can be utilized to improve the accuracy of fault diagnosis models and reduce training time. Therefore, the first level of transfer is the use of natural images as the source domain and screenshot images as the target domain while utilizing the VGG16 network that has been trained on the ImageNet dataset. Through model transfer, the fault diagnosis model is optimized with only a small number of screenshots to better extract the features of two-dimensional screenshot images. In order to avoid convolution operations and achieve real-time online diagnosis, the second level of transfer is the use of the features extracted from the screenshot images as the source domain and the one-dimensional sequence signals as the target domain. A DNN trained with the screenshot image features is used to optimize the DNN of the one-dimensional sequence signal, achieving the goal of integrating multi-source heterogeneous information. The specific steps are as follows.

Step 1. Based on the existing VGG16 network model, the CNN model of screenshots is optimized using model transfer, and the features of two-dimensional screenshots are extracted.

Using the existing VGG16 model trained on the ImageNet dataset, the features of monitoring screenshots are extracted through model transfer. Formula (1) represents the use of the trained VGG16 network and network parameters $P_{\mathrm{VGG}16}$ to extract the features $F_{2D}$ of screenshots $X_{2D}$. The two-dimensional features extracted in this study are the features of the first layer fully connected layer of VGG16.

(1) $$F_{2D}=G_{2D}(\mathrm{VGG}16,P_{\mathrm{VGG}16},X_{2D})$$

Step 2. A deep neural network model $DNN_{s}$ is built with screenshot image features as input.

(2) $$DNN_{s}=Feedforward(h_{s1},h_{s2},\cdots ,h_{sN})$$

(3) $$\left[DNN_{s},\theta_{s}^{\prime },{\tilde{\theta }}_{s}^{\prime }]=train(DNN_{s},F_{2D},\theta_{s},{\tilde{\theta }}_{s}\right)$$

As shown in Formula (2), a deep neural network model $DNN_{s}$ is established, which consists of N stacked self-encoders, and $h_{sj}$ represents the number of neurons in hidden layer j of $DNN_{s}$, j=1,2,…,N. Then, as shown in Formula (3), the network model $DNN_{s}$ is trained, in which the training set of $DNN_{s}$ is the screenshot feature $F_{2D}$ obtained in step 1. $\theta_{s}=\{\theta_{s1},\theta_{s2},\ldots ,\theta_{sN}\}$ represents the initial parameter set of N autoencoder coding networks, where $\theta_{sk}=\{W_{sk},b_{sk}\}$ represents the parameter set of the weight matrix and bias of the input layer and hidden layer of the kth autoencoder $AE_{k}$ in $DNN_{s}$, and these parameters are randomly initialized. ${\tilde{\theta }}_{s}=\{{\tilde{\theta }}_{s1},{\tilde{\theta }}_{s2},\ldots ,{\tilde{\theta }}_{sN}\}$ represents the initial parameter set of N autoencoder decoding networks, $\tilde{\theta }sk=\{{\tilde{W}}_{sk},d_{sk}\}$ represents the weight matrix and bias parameter set of $AE_{k}$’s hidden layer and output layer, and these parameters are also randomly initialized. The parameters of the $DNN_{s}$ model are trained and updated layer by layer, the updated encoding network parameters $\theta_{s}^{\prime }$ and decoding network parameters ${\tilde{\theta }}_{s}^{\prime }$ are obtained, and the abstract feature $H_{sN}=\sigma (W_{sN}^{\prime }\cdots (\sigma (W_{s2}^{\prime }(\sigma (W_{s1}^{\prime }X_{s}+b_{s1}^{\prime })+b_{s2}^{\prime }))+\cdots b_{sN}^{\prime })$ is obtained. The Softmax classifier is trained with $H_{sN}$ as input data, and the Softmax parameter $\theta_{ss}^{\prime }$ is updated and obtained. Finally, supervised backpropagation is used to fine-tune and optimize the parameters of $DNN_{s}$, and the fault diagnosis model $DNN_{s}$ and model parameter set $Ts=\{\theta_{s}^{\prime \prime },{\tilde{\theta }}_{s}^{\prime \prime },\theta_{ss}^{\prime \prime }\}$ trained using screenshot image features are obtained.

Step 3. A migration and integration network $DNN_{t}$ is established.

(4) $$DNN_{t}=Feedforward(h_{t1},h_{t2},\cdots ,h_{tN})$$

(5) $$\begin{array}{l} h_{t1}=h_{s1}\\ h_{t2}=h_{s2}\\ \;\;\;\vdots \\ h_{tN}=h_{sN} \end{array}$$

As shown in Formulas (4) and (5), a migration fusion network $DNN_{t}$ is established. In order to better fuse screenshot image features, except for the input layer, $DNN_{t}$ has the same network structure and the same number of neurons as $DNN_{s}$.

Step 4. The transfer from screenshot image features to one-dimensional sequence signals is realized.

The one-dimensional sequence signal and monitoring screenshot image are used to monitor the same object, but the storage form is different. In order to better integrate multi-source heterogeneous information and perform model transfer, the trained $DNN_{s}$ model with screenshot image features as input is used as the source domain, and the $DNN_{t}$ model with one-dimensional sequence signals as input is used as the target domain. By fusing screenshot image features, $DNN_{t}$ achieves better fault diagnosis results. The transfer is divided into three parts:

(1) Migration of encoding network parameters.

Due to the different input dimensions between the source and target domains, the structure of all DNN layers is the same, except for the input layer. The first-layer autoencoder coding network parameters $\theta_{t1}$ of the $DNN_{t}$ model are randomly initialized. As shown in Formula (6), the autoencoder coding network parameters $\theta_{s1}^{\prime \prime },\theta_{s2}^{\prime \prime },\ldots \ldots ,\theta_{sN}^{\prime \prime }$ from the second layer to the Nth layer of $DNN_{s}$ trained in step 2 are successively assigned to the coding network parameters $\theta_{t1},\theta_{t2},\ldots \ldots ,\theta_{tN}$ of the corresponding layer of $DNN_{t}$, and the initial parameter set of $DNN_{t}$’s coding network is $\theta_{t}=\{\theta_{t1},\theta_{t2},\ldots \ldots ,\theta_{tN}\}$.

(6) $$\begin{array}{l} \theta_{t2}=\theta_{s2}^{\prime \prime }\\ \;\;\;\vdots \\ \theta_{tN}=\theta_{sN}^{\prime \prime } \end{array}$$

(2) Migration of decoding network parameters.

Similarly to the migration of encoding network parameters, the first-layer decoding network parameter ${\tilde{\theta }}_{t1}$ of $DNN_{t}$ is randomly initialized. As shown in Formula (7), the decoding network parameters ${\tilde{\theta }}_{s1}^{\prime \prime },{\tilde{\theta }}_{s2}^{\prime \prime },\ldots \ldots ,{\tilde{\theta }}_{sN}^{\prime \prime }$ of the second to nth layers of the screenshot image feature $DNN_{s}$ trained in step 2 are sequentially assigned to the decoding network parameters ${\tilde{\theta }}_{t1},{\tilde{\theta }}_{t2},\ldots \ldots ,{\tilde{\theta }}_{tN}$ of the corresponding layers of $DNN_{t}$, and the initial parameter set of the decoding network of $DNN_{t}$ is ${\tilde{\theta }}_{t}=\{{\tilde{\theta }}_{t1},{\tilde{\theta }}_{t2},\ldots \ldots ,{\tilde{\theta }}_{tN}\}$.

(7) $$\begin{array}{l} {\tilde{\theta }}_{t1}={\tilde{\theta }}_{s1}^{\prime \prime }\\ \;\;\;\vdots \\ {\tilde{\theta }}_{tN}={\tilde{\theta }}_{sN}^{\prime \prime } \end{array}$$

(3) Migration of Softmax layer parameters.

As shown in Formula (8), the initialization parameter $\theta_{ts}$ of the target task Softmax classifier is obtained.

(8) $$\theta_{ts}=\theta_{ss}^{\prime \prime }$$

Step 5. The optimized transfer fusion model $DNN_{t}$ is trained.

(9) $$\left[DNN_{t},\theta_{t}^{\prime },{\tilde{\theta }}_{t}^{\prime }]=train(DNN_{t},X_{1D},\theta_{t},{\tilde{\theta }}_{t}\right)$$

As shown in Formula (9), the one-dimensional sequence signal $X_{1D}$ is used as the training dataset, and the network parameters $\theta_{t}$ and ${\tilde{\theta }}_{t}$ obtained through the migration in Step 4 are used as the initial parameters to train the $DNN_{t}$ fusion model. The updated encoding network parameters $\theta_{t}^{\prime }$ and decoding network parameters ${\tilde{\theta }}_{t}^{\prime }$ are obtained, and the abstract feature $H_{tN}=\sigma (W_{tN}^{\prime }\cdots (\sigma (W_{t2}^{\prime }(\sigma (W_{t1}^{\prime }X_{1D}+b_{t1}^{\prime })+b_{t2}^{\prime }))+\cdots b_{tN}^{\prime })$ is obtained. $H_{tN}$ is used as input data, and $\theta_{ts}$, obtained from Step 4, is used as the initial parameter of the fusion model Softmax classifier. The fusion model Softmax classifier is trained, and the Softmax parameter $\theta_{ts}^{\prime }$ is updated and obtained. Finally, supervised backpropagation is used to fine-tune and optimize the $DNN_{t}$ parameter $\left\{\theta_{t}^{\prime },{\tilde{\theta }}_{t}^{\prime },\theta_{ts}^{\prime }\right\}$, and the fusion model parameter $Tt=\{\theta_{t}^{\prime \prime },{\tilde{\theta }}_{t}^{\prime \prime },\theta_{ts}^{\prime \prime }\}$ is obtained.

Step 6. Online fault diagnosis.

The optimized fusion model $DNN_{t}$ can be used for real-time online fault diagnosis through migration. The diagnostic results of online one-dimensional sequence signal sample $x_{1D,online}(t)$ at time t are shown in Formulas (10) and (11). result(t) is the judgment category for the output sample $x_{1D,online}(t)$ of the $DNN_{t}$ model.

(10) $$h_{\theta_{ts}}(x_{1D,online}(t))=\left[\begin{array}{l} p(label(t)=1\;|x_{1D,online}(t);\theta_{ts})\\ p(label(t)=2|x_{1D,online}(t);\theta_{ts})\\ \;\;\;\;\;\;\;\;\;\;\;\;\;\vdots \\ p(label(t)=K|x_{1D,online}(t);\theta_{ts}) \end{array}\right]=\frac{1}{\sum_{k=1}^{K}e^{\theta_{tsk}^{T}x_{1D,online}(t)}}\left[\begin{array}{l} e^{\theta_{ts1}^{T}x_{1D,online}(t)}\\ e^{\theta_{ts2}^{T}x_{1D,online}(t)}\\ \;\;\;\;\;\;\;\;\;\vdots \\ e^{\theta_{tsK}^{T}x_{1D,online}(t)} \end{array}\right]$$

(11) $$result(t)=\underset{k=1,2\cdots ,K}{\arg\max}\{p(t)=k|x_{1D,online}(t);\theta_{ts})\}$$

**Figure 3:**
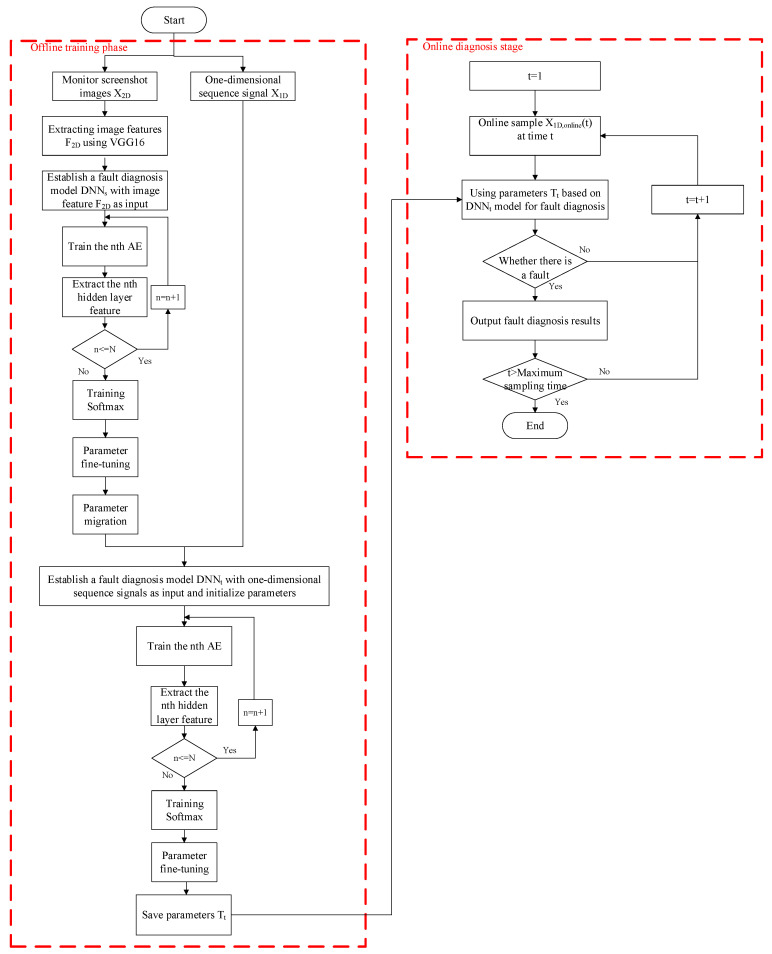
Flowchart of the multi-source heterogeneous information fusion fault diagnosis method based on two-level transfer learning.

The flowchart of the fault diagnosis method for multi-source heterogeneous information fusion based on two-level transfer learning is shown in Figure 3.

## 3. Time Complexity Analysis

This section analyzes the time complexity of the multi-source heterogeneous information fusion fault diagnosis algorithm based on two-level transfer learning. This study uses a stacked autoencoder to construct the DNN model. The DNN consists of four autoencoders and Softmax. A floating-point operation is recorded as one time unit. m represents the number of samples; *n_i_* is the number of neurons in the input layer of the DNN; *n*_1_, *n*_2_, *n*_3_, and *n*_4_, respectively, represent the number of neurons in the hidden layers of AE_1_, AE_2_, AE_3_, and AE_4_ that make up the DNN; s represents the number of types classified; l is the number of iterations; *n_b_* represents the batch size; and c is the time taken to calculate the gradient. As shown in Formulas (12)–(15), the time complexity of AE_1_, AE_2_, AE_3_, and AE_4_ is $O(m*n_{i}*n_{1}*l_{1})$, $O(m*n_{1}*n_{2}*l_{2})$, $O(m*n_{2}*n_{3}*l_{3})$, and $O(m*n_{3}*n_{4}*l_{4})$, respectively. The time complexity of Softmax and backpropagation is $O(n_{4}*s*l_{s})$ and $O(n_{b}*c*l_{b})$, respectively. As shown in Formula (16), the traditional DNN time complexity is $O(m*n^{2}*l)$.

(12) $$O(m*(n_{i}(n_{1}+1)+n_{1}(n_{i}+1))*l_{1})=O(m*n_{i}*n_{1}*l_{1})$$

(13) $$O(m*(n_{1}(n_{2}+1)+n_{2}(n_{1}+1))*l_{2})=O(m*n_{1}*n_{2}*l_{2})$$

(14) $$O(m*(n_{2}(n_{3}+1)+n_{3}(n_{2}+1))*l_{3})=O(m*n_{2}*n_{3}*l_{3})$$

(15) $$O(m*(n_{3}(n_{4}+1)+n_{4}(n_{3}+1))*l_{4})=O(m*n_{3}*n_{4}*l_{4})$$

(16) $$\begin{array}{l} O(m*n_{i}*n_{1}*l_{1}+m*n_{1}*n_{2}*l_{2}+m*n_{2}*n_{3}*l_{3}+m*n_{3}*n_{4}*l_{4}+n_{4}*s*l_{s}+n*c*l_{b})\\ =O(m*n^{2}*l) \end{array}$$

Then, the time complexity of the convolutional neural network is analyzed. Suppose that the size of the output feature graph of each convolution kernel is M*M, and *M* is determined by the input matrix X, the size K*K of each convolution kernel, the fill P, and the step size S, as shown in the Formula (17) [37].

(17) M=(X−K+2∗P)/S+1

The symbol I represents the number of input channels of each convolution kernel, that is, the number of output channels of the previous layer, and U represents the number of convolutional kernels of this convolution layer, that is, the number of output channels. The time complexity of a single convolutional layer is $O(M^{2}*K^{2}*I*U)$. Assuming that a convolutional neural network has a total of J convolutional layers, where j represents the *j*th convolutional layer and *U_j_* represents the number of convolutional kernels in j. The number of input channels *I*_j_ of the jth convolutional layer is equal to the number of output channels *U_j−1_* of the *j* − *1*th layer. The time complexity of forward extraction of image features using convolutional neural networks is $O(\sum_{j=1}^{J}M_{j}^{2}*K_{j}^{2}*U_{j-1}*U_{j})$ [38].

Fault diagnosis with multi-source heterogeneous information fusion based on two-level transfer learning is divided into offline training stage and online fault diagnosis stage, and a time complexity analysis is conducted for each stage separately.

The offline training process of the multi-source heterogeneous information fusion fault diagnosis algorithm based on two-level transfer learning mainly consists of four steps: 1. the VGG16 network model extracts screenshot image features; 2. the $DNN_{s}$ network is trained with screenshot image features as input; 3. transfer from the source model $DNN_{s}$ to the target model $DNN_{t}$ takes place; and 4. the target model $DNN_{t}$ is trained. The time complexity of Step 1 is $O(\sum_{j=1}^{J}M_{j}^{2}*K_{j}^{2}*U_{j-1}*U_{j})$. Step 2 is used to extract features by training and optimizing the stacked autoencoder. The time complexity of this stage is shown as $O(m*n^{2}*l)$ in Formula (16). Step 3 involves parameter migration, with a time complexity of O(n). The time complexity of Step 4 is the same as that of Step 2, which is $O(m*n^{2}*l)$. Due to the fact that the order of O(n) and $O(m*n^{2}*l)$ is smaller than that of $O(\sum_{j=1}^{J}M_{j}^{2}*K_{j}^{2}*U_{j-1}*U_{j})$, the time complexity of the offline training process is shown in Formula (18).

(18) $$O(\sum_{j=1}^{J}M_{j}^{2}*K_{j}^{2}*U_{j-1}*U_{j}+m*n^{2}*l+n+m*n^{2}*l)=O(\sum_{j=1}^{J}M_{j}^{2}*K_{j}^{2}*U_{j-1}*U_{j})$$

The online fault diagnosis process of the multi-source heterogeneous information fusion fault diagnosis algorithm based on two-level transfer learning involves inputting one-dimensional sequence signals and diagnosing them using the trained fault diagnosis model $DNN_{t}$. At this stage, there is no need to train and optimize the deep neural network. Only the trained deep neural network is used to extract features and output diagnostic results. The time complexity analysis of this stage is as follows: n_i_ is the number of neurons in the input layer of the DNN. n_1_, n_2_, n_3_, and n_4_ represent the number of neurons in the hidden layers of AE_1_, AE_2_, AE_3_, and AE_4_ that make up the DNN, and s represents the number of classification types. The time complexity of AE_1_, AE_2_, AE_3_, and AE_4_ is $O(n_{i}*n_{1})$, $O(n_{1}*n_{2})$, $O(n_{2}*n_{3})$, and $O(n_{3}*n_{4})$, respectively. The Softmax time complexity is $O(n_{4}*s)$. As shown in Formula (19), the time complexity of the online fault diagnosis process is $O(n^{2})$.

(19) $$O(n_{i}*n_{1}+n_{1}*n_{2}+n_{2}*n_{3}+n_{3}*n_{4}+n_{4}*s)=O(n^{2})$$

## 4. Experimental Verification

### 4.1. Experimental Analysis of Gearbox Data

#### 4.1.1. Data Description of Gearbox Data

**Figure 4:**
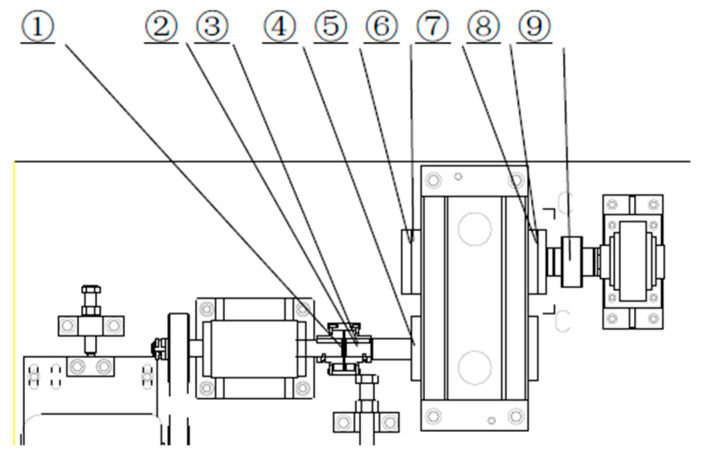
Schematic diagram of the gearbox test platform.

A gearbox is a complex piece of rotating mechanical equipment and a key component in systems for mechanical power transmission. It is widely used in industries such as petrochemicals, electricity, papermaking, and steel. Gears are important elements in gearboxes, and data show that two-thirds of gearbox failures are caused by gear failures. Once a gear malfunctions, the malfunction rapidly develops, affecting the normal operation of the equipment and causing huge economic losses. This study uses the gear fault dataset obtained from the QPZZ-II rotating machinery vibration test platform of Qianpeng Company. A schematic diagram of the test platform is shown in Figure 4, with the main parameters being a 0.75 kW motor, a maximum speed of 1470 r/min, three large gears with 75 teeth, and states of normal, pitted, and broken teeth, as well as two small gears with 55 teeth in normal and worn states. This experiment used three large gears and two small gears as experimental objects, with a speed of 1470 r/min and a sampling frequency of 5 KHz. The data collection work was completed with one photoelectric speed sensor, two displacement sensors, five acceleration sensors, and one electromagnetic speed sensor. The specific data collection, installation location, and meaning are shown in Table 1.

**Figure 5:**
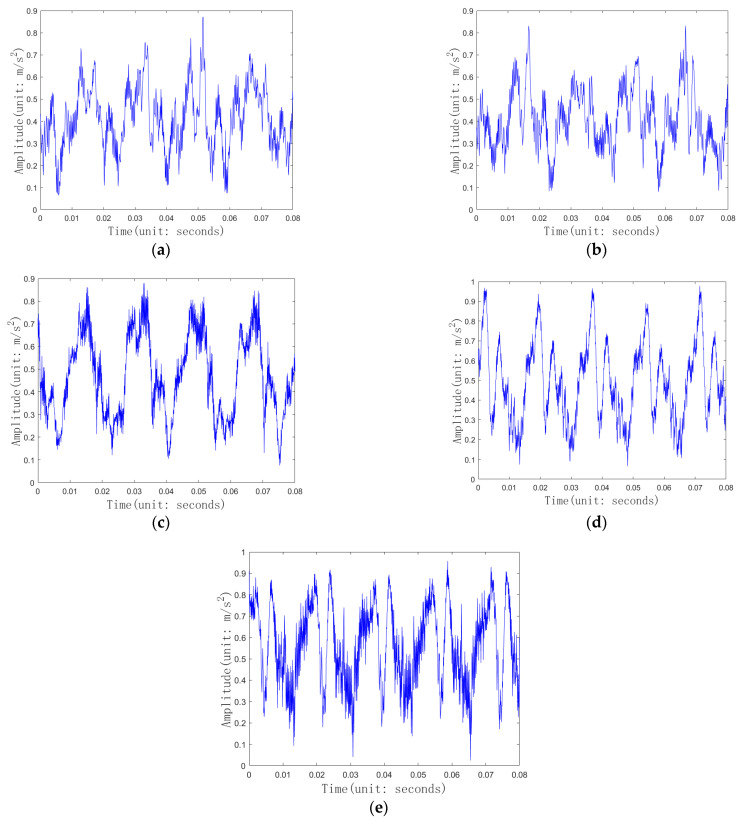
Screenshot image of photoelectric speed sensor monitoring: (**a**) normal; (**b**) pitting; (**c**) tooth breakage; (**d**) broken wear; (**e**) point wear.

There are five health states for gearboxes, namely, normal, large gear pitting (referred to as pitting), large gear tooth breakage (referred to as tooth breakage), large gear tooth breakage and small gear wear (referred to as broken wear), and large gear pitting and small gear wear (referred to as point wear). Assuming that the signals collected by the photoelectric speed sensor were monitored by the monitoring center monitor, the length of the slide box in the monitor screenshot of this experiment was set to 400, and the step size of the slide box was 20. That is, the two-dimensional image was the monitor screenshot image corresponding to the signals collected by the photoelectric speed sensor at 400 sampling points. The pixels of the two-dimensional image were 224 * 224, with a bit depth of 24. Figure 5 shows the screenshot images of the monitor for each health condition of the gearbox, which was the input of the convolutional neural network. The signals collected by nine sensors were used as one-dimensional signals, with each sample having a dimension of 9.

#### 4.1.2. Analysis of Experimental Results on the Gearbox Dataset

To verify the effectiveness of the proposed method, this section compares it with a DNN model using only one-dimensional signal sequence data, a CNN model using only monitor screenshot images, an FSS model using extended dimension fusion of one-dimensional signal sequence features and monitor screenshot image features, and a TVGG model using VGG16 for migration. The first comparison model (DNN) is a one-dimensional sequence signal collected by nine sensors, and the network structure is shown in Table 2. The second comparison model, CNN, only uses screenshot images from the center monitor. Its fault diagnosis model adopts a convolutional neural network, and the network structure and parameters are shown in Table 3. The third comparison model, FSS, concatenates the one-dimensional sequence features extracted from the first comparison model with the two-dimensional screenshot features extracted from the second comparison model. The concatenated features are used as inputs for the Softmax classifier to obtain diagnostic results. The fourth comparative model, TVGG, utilizes the VGG16 network trained on the ImageNet dataset to extract features from screenshot images, which are the features of the first fully connected layer of VGG16. Then, a deep neural network is used instead of the fully connected layer, and this feature is trained as the input of the deep neural network. The first-level migration of TTDNN has the same parameters as VGG16. The DNN model’s parameters used for the second-level migration are shown in Table 2. The DNN establishes a connection between the classifier and fault features, enabling the classifier to diagnose faults. If the number of hidden layers in the DNN is too small, the nonlinear descriptive ability is insufficient, which affects the diagnostic performance of the classifier. If the number of layers is too deep, it can cause overfitting problems, so four hidden layers are chosen. Neuron selection expands the dimension first, which enriches the feature representation. As the number of layers gradually increases, the number of neurons decreases, making the expression of fault features more refined.

In order to compare the differences in methods, this study compares not only the accuracy of fault diagnosis but also the online fault diagnosis time of various methods. In Formulas (20)–(24), t (x) represents the online fault diagnosis time of model x, G (x) represents the time required for model x to generate features, F (x) represents the time required for fault diagnosis model x to output diagnostic results, and S represents the time required for feature fusion. The first comparison model (DNN) is a fault diagnosis model trained only on one-dimensional sequence signals. As shown in Formula (20), the online fault diagnosis time is the time required for the online samples to be fed into the trained DNN model to output the diagnosis results. The second comparison model, the CNN, only uses screenshot images from the center monitor. As shown in Formula (21), the online fault diagnosis time is the time required for online samples to be fed into the trained CNN model to output diagnostic results. The diagnostic process of the third comparative model (FSS) is divided into four steps: 1. the monitor screenshot image is sent to the CNN model to extract the screenshot image features; 2. the one-dimensional sequence signal is sent into the DNN model to extract its features; 3. screen-captured image features and one-dimensional sequence signal features are concatenated; and 4. the concatenated features are sent to the Softmax classifier to output the diagnostic results. As shown in Formula (22), the online fault diagnosis time of FSS is the sum of these four steps. The diagnostic process of the fourth comparative model (TVGG) is divided into two steps: 1. the monitor screenshot image is sent to the VGG16 model to extract the screenshot image features; 2. the screenshot image features are sent into the DNN model to output the diagnostic results. The online fault diagnosis time of TVGG is shown in Formula (23). Since TTDNN has already transferred the screenshot image features to the DNN model, its input is a one-dimensional sequence signal, as shown in Formula (24). Its online fault diagnosis time is the time required for the online samples to be sent to the optimized DNN model for output diagnosis results.

t(DNN) = F(DNN)(20)

t(CNN) = F(CNN)(21)

t(FSS) = G(CNN) + G(DNN) + S + F(Softmax)(22)

t(TVGG) = G(VGG16) + F(DNN)(23)

t(TTDNN) = F(DNN)(24)

For the sake of fairness, after each model was trained, the model parameters were saved to the same computer for the comparison of the running time of each model. The computer model was a ThinkPad T450, which was manufactured by Lenovo in Beijing, China. The detailed parameters are shown in Table 4.

**Figure 6:**
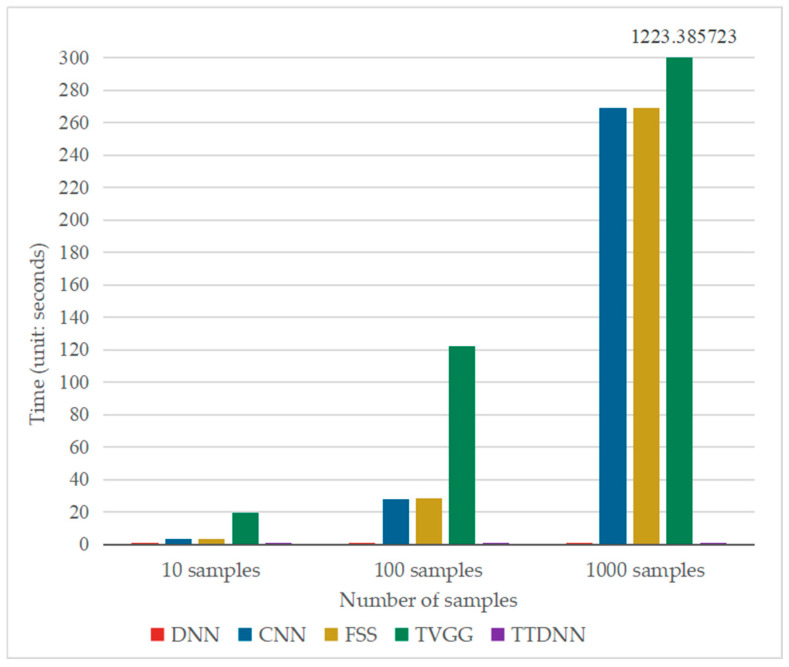
Bar chart of online diagnosis time for various models on the gearbox dataset.

Table 5 and Figure 6 show the running time of these five methods when the online data of the gearbox were 10, 100, and 1000. The running time details of the FSS and TVGG methods are shown in Table 6 and Table 7, respectively. Table 5 and Figure 6 show that when the online samples were 10, 100, and 1000, the online fault diagnosis time of the DNN and TTDNN was roughly the same, and they were less time-consuming than other methods. As shown in Table 6, the fault diagnosis time of FSS was the sum of four parts: CNN-generated features, DNN-generated features, feature concatenation, and the output of the fault diagnosis results. When the number of online samples is 10, the time for the CNN to generate features accounts for 91.96% of the FSS fault diagnosis time. When the number of online samples is 100, the time for the CNN to generate features accounts for 98.77% of the FSS fault diagnosis time. When the number of online samples is 1000, the time for the CNN to generate features accounts for 99.81% of the FSS fault diagnosis time. The fault diagnosis time of FSS is mainly determined by the time when the CNN generates features. As the number of online samples increases, the proportion of CNN-generated feature time to FSS fault diagnosis time increases. As shown in Table 7, when the number of online samples is 10, the time for VGG16 to generate features accounts for 98.14% of the TVGG fault diagnosis time. When the number of online samples is 100, the time for VGG16 to generate features accounts for 99.69% of the TVGG fault diagnosis time. When the number of online samples is 1000, the time for VGG16 to generate features accounts for 99.96% of the TVGG fault diagnosis time. The fault diagnosis time of TVGG is mainly determined by the time when VGG16 generates features, which accounts for more than 98% of the total fault diagnosis time of TVGG. The online fault diagnosis time of the five methods can be ranked as follows: TTDNN ≈ DNN < CNN < FSS < TVGG. When there are 1000 online samples, the online fault diagnosis time of the CNN is 757.20 times that of TTDNN, the online fault diagnosis time of FSS is 758.12 times that of TTDNN, and the online fault diagnosis time of TVGG is 3444.91 times that of TTDNN. From this, it can be seen that the CNN, FSS, and TVGG cannot achieve real-time fault diagnosis because they use convolutional neural networks for fault diagnosis. Convolutional neural networks have high time complexity and long computation time for a large number of convolution operations, which seriously affects the real-time performance of fault diagnosis.

In order to verify the effectiveness of the method proposed in this study, nine sets of experiments were conducted to analyze the diagnostic accuracy of each model based on the length of the sliding window and the number of screenshot images. The experimental results are shown in Table 8.

**Figure 7:**
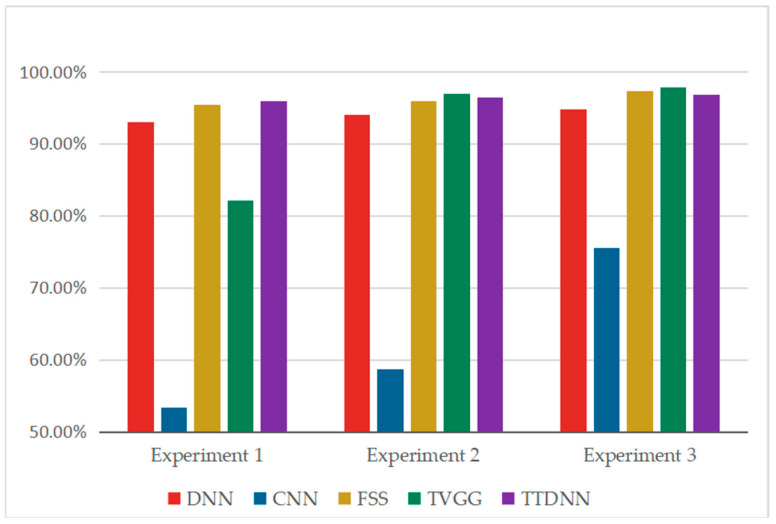
Bar chart of the fault diagnosis accuracy for various models on the gearbox dataset.

In experiments one to three, the length of the sliding window for the monitor screenshot images is 200, with a step size of 20. When the number of training samples for each class in the screenshot image is 20, 60, and 100, the corresponding number of training samples for each class in the one-dimensional sequence signal is 600, 1400, and 2200, respectively. The number of samples for each category in the test set is 200. With the increase in the training sample size, the fault diagnosis accuracy of each model is improved. The experimental results are shown in Figure 7. In the three experiments, compared with the DNN, although the time complexity of the DNN in the online diagnosis stage is the same as that of TTDNN, the fault diagnosis accuracy of the DNN is 2.00%–2.80% lower than that of TTDNN. TTDNN has a fault diagnosis accuracy that is 21.30%–42.60% higher than that of the CNN. Moreover, when there are 1000 online samples, the CNN’s online fault diagnosis time is 757.20 times that of TTDNN. It can be seen that when the number of screenshot images is small, convolutional neural networks trained only on screenshot images have poor performance. When the online sample size is 1000, the online fault diagnosis time of FSS is 758.12 times that of TTDNN. In the first two experiments, TTDNN had higher fault diagnosis accuracy than FSS, while in experiment 3, TTDNN had slightly lower fault diagnosis accuracy than FSS. It can be seen that when the number of screenshot images is small, TTDNN has better diagnostic performance than FSS. With the increase in the number of screenshot images, the fault diagnosis accuracy of TVGG significantly improves, and it is higher than that of TTDNN. However, the time complexity of TVGG’s online diagnosis stage is too high. When there are 1000 online samples, the online fault diagnosis time of TVGG is 3444.91 times that of TTDNN, and TVGG cannot achieve real-time fault diagnosis.

In experiments four to six, the length of the sliding window for the monitor screenshot image was 400, with a step size of 20. When the number of training samples for each class in the screenshot image was 20, 60, and 100, the corresponding number of training samples for each class in the one-dimensional sequence signal was 800, 1600, and 2400, respectively. The number of samples for each category in the test set was 200. The results of experiments four to six are basically consistent with the results of experiments one to three. The online diagnosis time of the DNN and TTDNN is the same, but the fault diagnosis accuracy of the DNN is lower than that of TTDNN. The fault diagnosis accuracy of the CNN is the worst, and the time complexity is high; thus, it cannot achieve the goal of real-time fault diagnosis. In experiment 4, the fault diagnosis accuracy of TTDNN was higher than that of FSS and TVGG, and the online fault diagnosis time of TTDNN was much shorter than that of FSS and TVGG. Therefore, when the number of screenshot images is small, the fault diagnosis effect of TTDNN is better.

In experiments seven to nine, the length of the sliding window for the monitor screenshot image was 600, with a step size of 20. When the number of training samples for each class in the screenshot image was 20, 60, and 100, the corresponding number of training samples for each class in the one-dimensional sequence signal was 1000, 1800, and 2600, respectively. The number of samples for each category in the test set was 200. The results of experiments seven to nine are consistent with those of experiments one to six. The number of screenshot training samples in experiments 1, 4, and 7 is the same, but the size of the sliding window is different. It can be seen that the larger the sliding window, the more information it contains, and the higher the accuracy of fault diagnosis.

The experimental results show that TTDNN has the same online diagnostic time as the DNN, and TTDNN has higher diagnostic accuracy than the DNN. The online diagnosis time of TTDNN is lower than that of the CNN, and the diagnostic accuracy of TTDNN is significantly higher than that of the CNN. When the number of screenshot images is very small, the fault diagnosis accuracy of TTDNN is higher than that of FSS and TVGG, and the online fault diagnosis time of TTDNN is much shorter than that of FSS and TVGG. As the number of screenshot images increases, the FSS and TVGG methods have higher diagnostic accuracy than TTDNN. However, due to the high time complexity of FSS and TVGG, real-time online diagnosis cannot be performed.

### 4.2. Experimental Analysis of Bearing Data

#### 4.2.1. Data Description of Bearing Data

**Figure 8:**
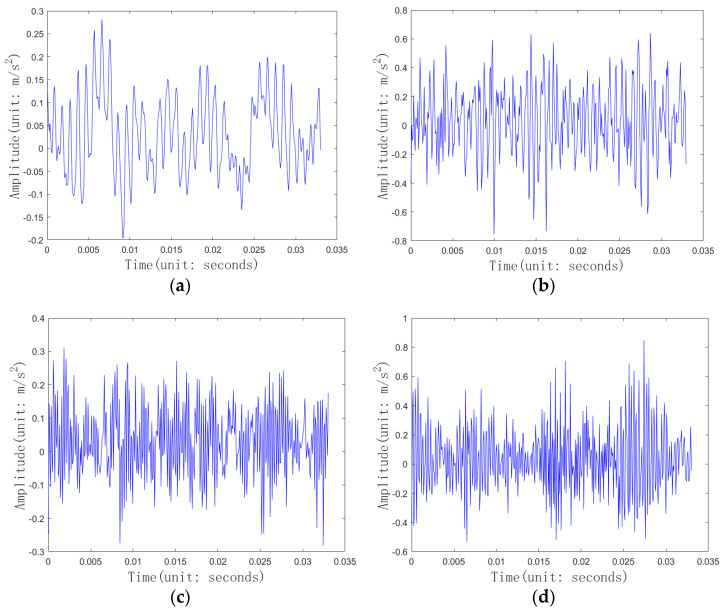
Screenshot image of fan end monitoring: (**a**) normal; (**b**) inner ring fault; (**c**) ball bearing fault; (**d**) outer ring fault.

As an indispensable component in large-scale manufacturing production, the health status of bearings directly affects the stable operation of entire systems. In this study, experiments were conducted using the bearing dataset from Case Western Reserve University [39], which is widely used in the field of fault diagnosis. The fault diameters provided by this dataset are 0.007 inches, 0.014 inches, 0.021 inches, and 0.028 inches. The motor load has four states, 0 hp, 1 hp, 2 hp, and 3 hp, and the motor speed has four states, 1797 RPM, 1772 RPM, 1750 RPM, and 1730 RPM. There are two sampling frequencies: 12 kHz and 48 kHz. The data used in this section are bearing monitoring data with a fault diameter of 0.007 inches, a motor load of 0 Hp, a motor speed of 1797 RPM, and a sampling frequency of 12 KHz. This section uses the vibration signals of the bearings at the motor drive end and fan end as experimental data. The vibration signals of the bearings at the motor drive end and fan end are used as one-dimensional signals, while the fan end is monitored by the center monitor to obtain two-dimensional screenshot images. The health status of bearings can be divided into four types: the normal state, inner ring fault, ball bearing fault, and outer ring fault. The length of the sliding frame in this experiment is set to 400, and the step size of the sliding frame is 20. The two-dimensional screenshot image is a monitor screenshot image corresponding to 400 vibration signals at the fan end. The two-dimensional screenshot image is synchronized with the one-dimensional sequence, and the one-dimensional sequence signal samples are 400 bearing vibration signals at the motor drive end or fan end. The pixel size of the 2D screenshot image is 224 * 224, with a bit depth of 24. Figure 8 shows screenshot images of the monitoring of the fan end for each healthy state of the bearing, which is the input of the convolutional neural network.

#### 4.2.2. Analysis of Experimental Results on the Bearing Dataset

The methods used for comparison in this section are the same as those in Section 4.1.2. After training each model, the model parameters were saved to the same computer to compare the running time of each model. The computer model was a ThinkPad T450, and the detailed parameters are shown in Table 4.

**Figure 9:**
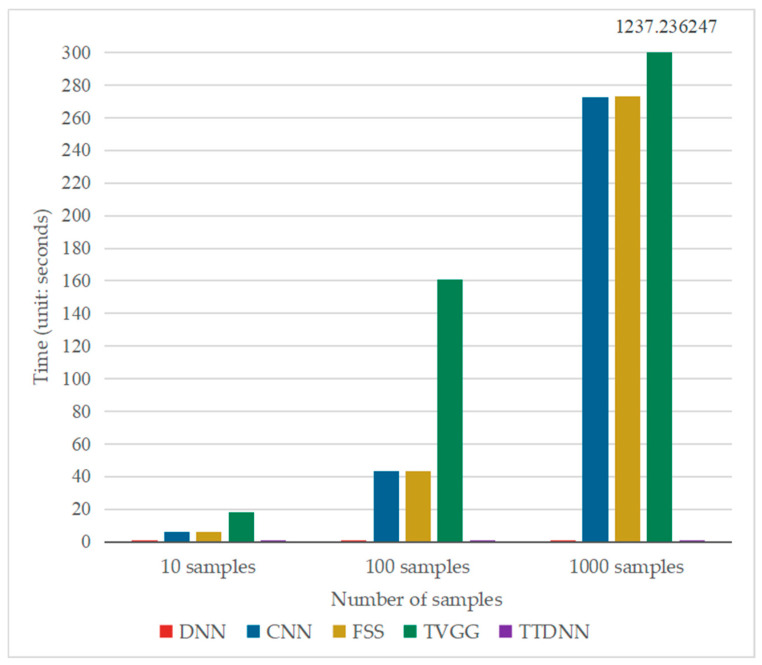
Bar chart of the online diagnosis time for various models on the bearing dataset.

Table 9 and Figure 9 show the running time of these five methods when there were 10, 100, and 1000 samples, and the running time details of the FSS and TVGG methods are shown in Table 10 and Table 11, respectively. As shown in Table 10, when the number of online samples is 10, the time for the CNN to generate features accounts for 95.89% of the FSS fault diagnosis time. When the number of online samples is 100, the time for the CNN to generate features accounts for 99.21% of the FSS fault diagnosis time. When the number of online samples is 1000, the time for the CNN to generate features accounts for 99.82% of the FSS fault diagnosis time. The fault diagnosis time of FSS is mainly determined by the time when the CNN generates features. As the number of online samples increases, the proportion of CNN-generated feature time to FSS fault diagnosis time increases. As shown in Table 11, when the number of online samples is 10, the time for VGG16 to generate features accounts for 98.25% of the TVGG fault diagnosis time. When the number of online samples is 100, the time for VGG16 to generate features accounts for 99.78% of the TVGG fault diagnosis time. When the number of online samples is 1000, the time for VGG16 to generate features accounts for 99.95% of the TVGG fault diagnosis time. The fault diagnosis time of TVGG is mainly determined by the time when VGG16 generates features, which accounts for more than 98% of the total fault diagnosis time of TVGG. The online diagnosis time of each model is consistent with the results in Section 4.1, and the online fault diagnosis time of the five methods is ranked as follows: TTDNN ≈ DNN < CNN < FSS < TVGG. When there are 1000 online samples, the online fault diagnosis time of the CNN is 757.75 times that of TTDNN, that of FSS is 758.55 times that of TTDNN, and that of TVGG is 3437.70 times that of TTDNN. The results indicate that the more convolutional layers there are in the convolutional neural network model, the longer the online fault diagnosis time, resulting in an inability to perform real-time online fault diagnosis. Only TTDNN and the DNN can meet the requirements of real-time online fault diagnosis.

**Figure 10:**
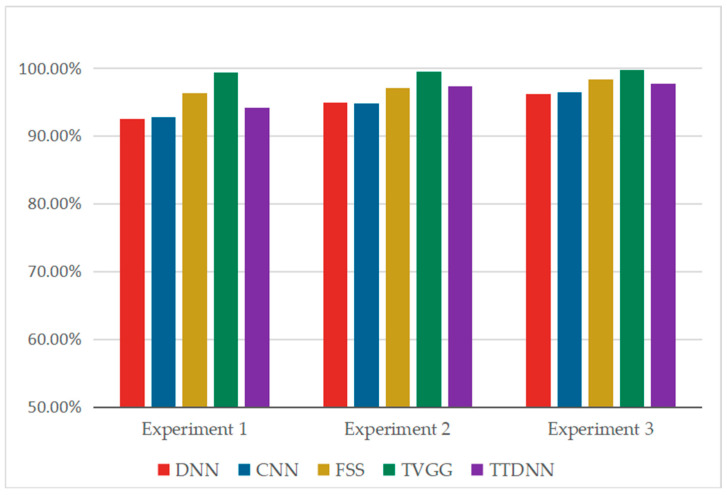
Bar chart of fault diagnosis accuracy for various models on the bearing dataset.

In this section, three sets of experiments were conducted. The number of training samples for each class of one-dimensional sequence signals was 100, 120, and 140, respectively. Since the sliding window and step size of the screenshot image were the same as those of the one-dimensional sequence signal, the number of training samples for each class of the screenshot image was also 100, 120, and 140, respectively. The number of samples for each category in the test set was 200. The experimental results are shown in Table 12 and Figure 10. With the increase in the training sample size, the fault diagnosis accuracy of each model is improved. Due to the larger number of screenshot images in this section compared with Section 4.1, the fault diagnosis accuracy of the CNN is significantly improved, but it is still lower than that of the TTDNN method proposed in this study, and the online diagnosis time of TTDNN is much shorter than that of the CNN. Although the time complexity of the DNN in the online diagnosis stage is the same as that of TTDNN, the fault diagnosis accuracy of the DNN is 1.50–2.38% lower than that of TTDNN, indicating that TTDNN integrates the features of screenshot images and improves the fault diagnosis accuracy of deep neural networks. As the number of screenshot images increases, the fault diagnosis accuracy of FSS and TVGG becomes higher than that of TTDNN. However, the online fault diagnosis time of FSS and TVGG is longer and cannot meet the needs of online fault diagnosis.

## 5. Conclusions

To solve the problem of poor real-time performance of fault diagnosis algorithms when multi-source heterogeneous information and external domain data are fully utilized, a multi-source heterogeneous information fusion fault diagnosis method (TTDNN) based on two-level transfer learning is proposed in this study. This method realizes the migration from a convolutional neural network to a deep neural network by establishing migration between deep learning models based on heterogeneous information from multiple sources. The fault diagnosis model with two-level transfer not only integrates the features of one-dimensional sequence signals and screenshot images but also avoids convolution operations and has a low time complexity. The effectiveness of the proposed method was verified using experiments on a gearbox dataset and bearing dataset.

The samples from the source domain in this study were not screened and were transferred to the target domain as a whole. However, some samples in the source domain may have a negative impact on the target domain, so future work should consider using the filtering of irrelevant source data before migration to remove source domain samples that have a negative impact on the target domain. This can avoid negative migration, improve migration effectiveness, and, ultimately, enhance the accuracy of fault diagnosis in the target domain. This study adopted model transfer, which can be used to further study the combination of domain adaptation and model transfer. By adding an adaptive layer between the feature extraction layer and the classification layer, the distribution distance between the source domain and the target domain is minimized, thereby enabling the model to obtain a powerful classifier. Furthermore, fault diagnosis methods based on domain adaptation and model transfer can be compared.

## Figures and Tables

**Table 1:** Sensor channels, their installation locations, and their meanings.

Channel	Installation Position Corresponding to Figure 4	Sensor
CH1	①	Photoelectric speed sensor
CH2	②	Horizontal displacement of the input axis
CH3	③	Vertical displacement of input axis
CH4	④	Input shaft motor side bearing acceleration
CH5	⑤	Horizontal acceleration of the output shaft motor side bearing
CH6	⑥	Vertical acceleration of the output shaft motor side bearing
CH7	⑦	Horizontal acceleration of the load side bearing on the output shaft
CH8	⑧	Vertical acceleration of the load side bearing on the output shaft
CH9	⑨	Output shaft load side bearing magneto electric speed

**Table 2:** Parameters of the comparative fault diagnosis model (DNN).

Hidden Layers	Number of Neurons in Each Hidden Layer	Maximum Number of Iterations	Learning Rate	Momentum Factor
4	600/400/200/100	1000	0.1	0.05

**Table 3:** CNN fault diagnosis model parameters.

Layer Name	Layer Parameters
Input	224 * 224 * 3
Convolutional layer 1	Convolutional kernels 11 * 11 * 3, number of convolution kernels: 16
Pooling layer 1	7 * 7 maximum pooling
Convolutional layer 2	Convolutional kernels 11 * 11 * 3, number of convolution kernels: 32
Pooling layer 2	7 * 7 maximum pooling
Fully connected layer 1	512
Fully connected layer 2	138

**Table 4:** Detailed parameters of the computer.

Name	Specification
Processor	Intel Core i5-5200U CPU@2.20 GHz
Main memory	8.00 GB
Graphics card	Intel(R) HD Graphics 5500
Operating system	Windows 10

**Table 5:** Comparison of the online diagnosis time for various models on the gearbox dataset (unit: seconds).

Number of Samples	DNN	CNN	FSS	TVGG	TTDNN
10	0.287781	3.223261	3.479948	19.758033	0.278431
100	0.295396	27.977264	28.166509	121.899118	0.309738
1000	0.325549	268.904582	269.231330	1223.385723	0.355129

**Table 6:** Online diagnosis time of FSS on the gearbox dataset (unit: seconds).

Number of Samples	G (CNN)	G (DNN)	S	F (Softmax)	Total Time
10	3.200159	0.014895	0.000143	0.264751	3.479948
100	27.821015	0.023527	0.000299	0.321668	28.166509
1000	268.723600	0.106404	0.001693	0.399633	269.231330

**Table 7:** Online diagnosis time of TVGG on the gearbox dataset (unit: seconds).

Number of Samples	G (VGG16)	F (DNN)	Total Time
10	19.391099	0.366934	19.758033
100	121.519353	0.379765	121.899118
1000	1222.844465	0.541258	1223.385723

**Table 8:** Results of the comparative experiments on the gearbox dataset.

Diagnostic Accuracy	DNN	CNN	FSS	TVGG	TTDNN
Experiment 1	93.10%	53.30%	95.40%	82.10%	95.90%
Experiment 2	94.00%	58.70%	95.90%	97.00%	96.50%
Experiment 3	94.80%	75.50%	97.30%	97.90%	96.80%
Experiment 4	93.20%	61.90%	95.80%	83.70%	96.10%
Experiment 5	94.10%	76.90%	97.00%	98.20%	96.70%
Experiment 6	95.10%	80.10%	98.00%	98.80%	97.00%
Experiment 7	93.90%	62.60%	95.90%	94.20%	96.40%
Experiment 8	94.30%	78.10%	97.10%	98.70%	96.80%
Experiment 9	95.60%	80.20%	98.10%	99.80%	97.40%

**Table 9:** Comparison of the online diagnosis time for various models on the bearing dataset (unit: seconds).

Number of Samples	DNN	CNN	FSS	TVGG	TTDNN
10	0.279155	5.957512	6.142723	18.370224	0.281735
100	0.298802	43.316780	43.540302	160.834970	0.307586
1000	0.362661	272.716783	273.005223	1237.236247	0.359902

**Table 10:** Online diagnosis time of FSS on the bearing dataset (unit: seconds).

Number of Samples	G (CNN)	G (DNN)	S	F (Softmax)	Total Time
10	5.890179	0.015517	0.000160	0.236867	6.142723
100	43.197073	0.064924	0.000336	0.277969	43.540302
1000	272.524246	0.114095	0.001393	0.365489	273.005223

**Table 11:** Online diagnosis time of TVGG on the bearing dataset (unit: seconds).

Number of Samples	G (VGG16)	F (DNN)	Total Time
10	18.048359	0.321865	18.370224
100	160.486914	0.348056	160.834970
1000	1236.666685	0.569562	1237.236247

**Table 12:** Results of the comparative experiment on the bearing dataset.

Diagnostic Accuracy	DNN	CNN	FSS	TVGG	TTDNN
Experiment 1	92.50%	92.75%	96.38%	99.38%	94.25%
Experiment 2	95.00%	94.87%	97.13%	99.50%	97.38%
Experiment 3	96.25%	96.50%	98.38%	99.75%	97.75%

## Data Availability

The data involved in this article have been presented in this article.
